# Peer Review of “Impact of the COVID-19 Pandemic on the Mental Health of College Students in India: Cross-sectional Web-Based Study”

**DOI:** 10.2196/32952

**Published:** 2021-09-02

**Affiliations:** 1 NA NA, ON Canada

*This is a peer-review report submitted for the paper “Impact of the COVID-19 Pandemic on the Mental Health of College Students in India: Cross-sectional Web-Based Study”.*

## Round 1 Review

### General comments

This paper [1] is timely given the situation in India. Unfortunately, the manuscript does not include information that can help the readers to contextualize the need to do the study and the rationale to conduct the validation. The discussion is too general to raise awareness concerning the mental health of university students.
